# Relationship between folate concentration and expression of folate-associated genes in tissue and plasma after intraoperative administration of leucovorin in patients with colorectal cancer

**DOI:** 10.1007/s00280-018-3690-9

**Published:** 2018-09-29

**Authors:** Helena Taflin, Elisabeth Odin, Kristoffer Derwinger, Göran Carlsson, Bengt Gustavsson, Yvonne Wettergren

**Affiliations:** Department of Surgery, Institute of Clinical Sciences, Sahlgrenska University Hospital/Östra, Sahlgrenska Academy at University of Gothenburg, 41685 Gothenburg, Sweden

**Keywords:** Folate, Colorectal cancer, Gene expression, PCFT, RFC-1

## Abstract

**Purpose:**

The aim of study was to investigate the relationship between folate concentration and expression of folate-associated genes in tumour, mucosa and plasma of patients with colorectal cancer, after intraoperative administration of bolus leucovorin (LV).

**Methods:**

Eighty patients were randomized into four groups to receive 0, 60, 200, or 500 mg/m^2^ LV, respectively. Tissue and plasma folate concentrations were assessed by LC–MS/MS. Gene expression of ABCC3/MRP3, FPGS, GGH, MTHFD1L, SLC46A1/PCFT, and SLC19A1/RFC-1 was determined using quantitative PCR.

**Results:**

The folate concentration in tumour increased with increasing dosage of LV. Half of the patients treated with 60 mg/m^2^ did not reach a level above the levels of untreated patients. A significant correlation between folate concentration in tumour and mucosa was found in untreated patients, and in the group treated with 60 mg/m^2^ LV. The 5-MTHF/LV ratio correlated negatively with folate concentration in mucosa, whereas a positive correlation was found in tumour of patients who received 200 or 500 mg/m^2^ LV. A positive correlation was found between folate concentration and expression of all genes, except MTHFD1L, in patients who received LV. There was a negative correlation between 5-MTHF concentration in plasma of untreated patients and expression of GGH and SLC46A1/PCFT in tumour.

**Conclusions:**

The results indicate the possibility of using the individual plasma 5-MTHF/LV ratio after LV injection as a surrogate marker for tissue folate concentration. Expression of several folate-associated genes is associated with folate concentration in tissue and plasma and may become useful when predicting response to LV treatment.

**Electronic supplementary material:**

The online version of this article (10.1007/s00280-018-3690-9) contains supplementary material, which is available to authorized users.

## Introduction

Colorectal cancer (CRC) is a major cause of cancer death worldwide [1, 2]. The median overall survival from metastatic disease is now reaching almost 30 months in clinical trials, which is due to development of new biological agents in addition an increasing number of patients undergoing surgical resection of localized metastatic disease. A more strategic approach to the delivery of systemic therapy and an expansion in the use of ablative techniques may also contribute to improvement of survival [3]. However, in the clinical setting, the overall survival can be expected to be lower [4].

The fundament in the treatment of CRC is surgery. However, after primary surgery, treatment with chemotherapy is recommended for advanced tumour stages [5]. The drug 5-fluorouracil (5-FU) is used as a cornerstone of chemotherapy treatment, in the adjuvant, as well as in the palliative setting for CRC [6–8]. This drug is an analogue of uracil, in which the hydrogen at position 5 is replaced by fluorine. 5-FU enters the cell in the same way as uracil and is then converted in several steps intracellularly to the final active metabolite 5-fluoro-2′-deoxyuridine monophosphate (FdUMP). The enzyme thymidylate synthase (TS; EC 2.1.1.45), catalyses the reductive methylation of deoxyuridine monophosphate (dUMP) to deoxythymidine monophosphate (dTMP) using 5,10-methylenetetrahydrofolate (5,10-MeTHF) as the methyl-group donor. Subsequently, dTMP is further phosphorylated to become deoxythymidine triphosphate (dTTP), which is an essential nucleotide needed in DNA synthesis and repair. When FdUMP is replacing dUMP, the TS enzyme is inhibited thereby blocking the conversion of dUMP to dTMP. Since this conversion is the only way for de novo formation of dTMP, the blockage will impair both DNA synthesis and repair [8, 9]. The greatest impact will be on cells having a high proliferation rate, such as cancer cells [10].

The response rate of colorectal tumours to 5-FU monotherapy is only around 10%. By adding a reduced folate, the tumour response rate can be improved to 21%, as has been shown in a meta-analysis [11]. In clinical practice, patients treated with 5-FU will receive the reduced folate leucovorin (LV) in the form of a stable calcium salt of 5-formyltetrahydrofolic acid (calcium folinate). After injection, LV is transported to the liver where it is mainly metabolized to 5-methyltetrahydrofolate (5-MTHF). This metabolite is then transported to the blood stream to reach other tissues. The maximum plasma concentration of 5-MTHF is reached 30 min after i.v. administration [12]. LV is eliminated by 80–90% through the kidneys.

The Nordic FLV therapy, which is a regime of bolus 5-FU and LV that was introduced in the 1990s, is still the cornerstone of both adjuvant and palliative treatments for CRC in Nordic countries [13]. Although the Nordic FLV is a well-established regimen, the evidence for a beneficial effect of the LV dosage and administration used is rather limited. Different regimens used in clinical practice worldwide apply LV concentrations that range from 20 to 500 mg/m^2^ and are often empirically evolved [14].

Leucovorin needs to be converted in several steps into the active substance 5,10-MeTHF. This metabolite is polyglutamated by the enzyme folylpolyglutamate synthase (FPGS), which increases its cellular retention. 5,10-MeTHF stabilizes the ternary complex between TS and FdUMP, hence, leading to inhibition of the formation of dTMP from dUMP [15]. In order to get a complete inhibition of TS, the active metabolite 5,10-MeTHF must be in excess. Then, a maximal formation of the ternary complex consisting of TS, FdUMP, and 5,10-MeTHF can be achieved [16, 17].

A recent article published by our group presented data showing a large inter-individual variation of tissue folate concentration in patients with CRC after supplementation with LV at standardized dosage [18]. The concentrations of tetrahydrofolate (THF), 5,10-MeTHF and 5-MTHF in tumour and mucosa were assessed by liquid chromatography electrospray ionization tandem mass spectrometry (LC–MS/MS). The results showed that the 5,10-MeTHF concentration in tumours of many patients given the standardized dose of LV, i.e. 60 mg/m^2^, did not exceed the values of an untreated control group. Rectal cancer patients in particular required high doses of LV to reach a tumour tissue concentration of 5,10-MeTHF above baseline. Similar results have been described by Houghton et al. [19].

In the present study, we hypothesize that folate administration has an effect on gene expression that may be clinically relevant. The folate concentration in matching tumour and mucosa tissue obtained from patients with CRC were determined and compared with the concentration in plasma after treatment with increasing doses of LV. The impact of the folate concentration in tissue and plasma on gene expression of six folate pathway genes was analysed.

## Patients and methods

Eighty patients scheduled for a colorectal resection with a cancer indication were enrolled in the study between January 2011 and January 2012. All patients gave their written informed consent. The pre-operative exclusion criteria were patient inability to understand the study information or inability to provide true informed consent. There were no other exclusion criteria. The patients were pre-operatively randomized into four groups; the first served as control group and received no LV. Groups 2, 3, and 4 received 60, 200, and 500 mg/m^2^ LV, respectively, administered intravenously as a bolus injection at the initiation of general anaesthesia. The LV was manufactured in the form of calcium folinate (RS-LV) supported by Teva Sweden AB, Helsingborg, Sweden. The surgeon was blinded to the dosage given and all patients were otherwise treated in accordance with normal routines and guidelines.

During surgery, at the time of removal of the surgical specimen, a research nurse collected fresh tissue samples from both tumour and macroscopically normal-appearing mucosa located 10 cm from the tumour. The biopsies were snap-frozen in liquid nitrogen and stored at − 80 °C until used. Clinical and pathology data regarding diagnosis, tumour differentiation and stage, and pre-operative treatment regimen were retrieved to assess the different groups and enable a better understanding of the factors that might influence treatment responses.

### Determination of plasma folate

Blood samples were obtained from all patients. The samples were collected at 0, 10, and 30 min. in EDTA vacutainers and immediately centrifuged (4 °C, 2000 g, 10 min). The plasma was stored at − 80 °C until LC–MS/MS analysis. To 1 ml of [(0,2% formic acid in acetonitrile): Methanol (9:1)], 200 µl plasma was added, and mixed well for 10 min. Aminoacetophenone was used as internal standard. Stock aminoacetophenone solution (0.1 mM) was stored at − 80 °C until use. After addition of 20 µl internal standard and 30 µl of water, the samples were centrifuged for 10 min at 21,500*g*. The supernatant was loaded on a 1 cc Oasis PRIME HLB Cartridge and passed-through. The eluate was collected and evaporated to dryness and reconstituted with 300 µl Mobile phase A before analysis. A stock solution (20 mM) of LV and 5-MTHF was dissolved in extraction buffer (50 mM phosphate buffer (pH 7.0), 1% sodium ascorbate and 0.1% β-mercaptopropanol) and stored at − 80 °C until use. The folate solution was serially diluted in extraction buffer to prepare the calibration curves. A blank plasma sample was used to dilute the standard samples. A mixture of standards and internal standard was extracted as described for the samples. Calibration standards containing ten different concentrations for LV and 5-MTHF were used. The quality (Q) controls low, medium and high were prepared in plasma, and extracted as described for the samples. The extracted ions following MRM transitions were monitored at *m/z* 460 → 313 for 5-MTHF, *m/z* 474 → 327 for LV, and 136 → 94 for aminoacetophenone. The mean calibration curves for 5-MTHF (*y* = 0.1883*x* + 0.0187, *R*^2^) and LV (*y* = 0.0752*x* + 0.1836, *R*^2^ = 0.9973) were measured on different days (*n* = 9). Variability was determined by analysing plasma Q-sample (*n* = 7) at low, medium and high concentration and also between days (*n* = 9). The relative standard deviation (RSD) for 5-MTHF and LV ranged from 2 to 6.2% within the same day and the variability over 9 days ranged from 4.9 to 10.4%. Sensitivity was assessed by evaluating the limit of detection (LOD) and limit of quantification (LOQ) for the method. The LOD and LOQ were defined as the lowest analyte concentration yielding a signal-to-noise (S/N) ratio of 3 and 10, respectively. The LOD for 5-MTHF and LV was 0.3 and 1.6 pmol/ml, respectively. The LOQ for 5-MTHF and LV was 0.9 was and 5.2 pmol/ml, respectively.

### Determination of tissue folate

LC–MS/MS was used to measure concentrations of the folate derivatives 5,10-MeTHF, THF, and 5-MTHF, expressed as pmol/g wet-weight (pmol/gww), in tumour tissue and adjacent mucosa [20]. Raltitrexed was used as an internal standard. The sum of the 5,10-MeTHF, THF, and 5-MTHF concentrations was used as a measure of folates in the tissue. The extracted ions following MRM transitions were monitored at *m/z* 446 → 299 for THF, *m/z* 458 → 311 for 5,10-MeTHF, *m/z* 460 → 313 for 5-MTHF, and *m/z* 459 → 312 for raltitrexed.

On the day of sample analysis, extraction buffer was prepared containing 50 mM phosphate buffer, pH 7.0, 1% ascorbate, and 0.1% β-mercaptopropanol. The tissue was weighed and placed in an Eppendorf vial and a 10 × volume of extraction buffer was added. Homogenization was performed using a TissueLyser (two disruption steps at 25 Hz for 2.5 min). After a deconjugation step, protein precipitation, centrifugation, and ultrafiltration (30 min at 21,500×*g* at 20 °C) were performed. The solution at the bottom of the test tube was used for LC–MS/MS analysis. The relative standard deviation (RSD) ranged from 2 to 7% for all analyses, and the variability over 4 days ranged from 3 to 14%. The accuracy of the method was determined by estimating the recovery by adding known amounts of the standard to a sample. The average recoveries were 98, 87, and 93% for THF, 5,10-MeTHF, and 5-MTHF, respectively. The LOD for 5,10-MeTHF, THF and 5-MTHF was 2.1, 1.2 and 0.3 pmol/g_ww_, respectively. The LOQ for 5,10-MeTHF, THF and 5-MTHF was 4.0, 7.0 and 0.9 pmol/g_ww_, respectively. Standard curves for 5,10-MeTHF, THF, and 5-MTHF in tissue have been presented in a previous paper [20].

The LC–MS/MS analyses were performed on a Waters 2795 LC separation module coupled to a Waters Micromass Quattro Triple-Quadrupole MS system with an electrospray ionization (ESI) source. Folates were detected and quantified using positive electrospray. The separation of folates was performed using an Atlantis dC18 3 µm, 2.1 × 100 mm column (Waters) together with the guard column Atlantis dC18, 3 µm, 2.1 × 10 mm. The mobile phase consisting of eluent A (0.1% of acetic acid in water) and eluent B (0.1% acetic acid in acetonitrile) was used. Calibration graphs were constructed by plotting the peak area ratio of each compound to internal standards against concentration. The standards and samples were processed using the QuanLynx quantitative processing tool in MassLynx (Waters Corp., Milford, MA, USA). A more detailed description of the folate assay development has been published previously [20].

### Preparation of RNA and cDNA

Total RNA was isolated from 10 to 30 mg fresh-frozen tissue using the High Pure RNA Tissue Kit (#12033674001, Roche Diagnostics Scandinavia AB) according to the manufacturer’s instructions. cDNA was synthesized using the High Capacity cDNA Reverse Transcription Kit (Applied Biosystems) and run on Gene Amp PCR System 9600 (Perkin Elmer). To optimize each run, the expression level of β-actin was determined in each sample. A second RNA extraction and cDNA synthesis was performed if the concentration was considered to be suboptimal.

### Real-time quantitative PCR

Based on recent studies [19], five target genes with putative impact on LV metabolism were chosen for analysis (Supplementary Table 1). These genes are involved in folate transport (ABCC3, SLC19A1/RFC-1 and SLC46A1/PCFT), folate polyglutamation (FPGS and GGH) or folate metabolism (MTHFD1L). The relative gene expression was quantified in tumours and mucosa using real-time quantitative PCR (qPCR). TaqMan Gene Expression Assays (Life Technologies, Stockholm, Sweden) were ordered for each gene from Applied Biosystems at http://www.appliedbiosystems.com. The qPCR was set up in triplicates in 384-well plates using a Nanodrop II (GC Biotech) and was carried out in 5 µl reactions with 1 × TaqMan^®^ Gene Expression Mastermix (Applied Biosystems), 1 × gene-specific TaqMan assay (Applied Biosystems), and 1 µl cDNA. The qPCR was run on a QuantStudioTM 12K Flex Real-Time PCR System (Life Technologies, Stockholm, Sweden) according to a standard protocol. The thresholds and baselines were set manually using the sequence detection systems software (SDS), version 2.4 (Applied Biosystems), and cycle threshold (*C*_t_) values were extracted. Variations between runs were compensated for by normalization against a control sample. There was a linear correlation between the two house-keeping genes ACTB and GAPDH. All *C*_t_ values were normalized to a mean value representing both of these genes in order to keep variance to the minimum.

### Statistical analyses

The JMP 11.0/SAS software (SAS Institute Inc. Cary, NC, USA) was used for the statistical analyses. All gene expression calculations were performed using the ΔΔ*C*_t_ method. As the values of the gene expression and folate concentrations were not normally distributed, they were transformed to be logarithmic. Differences between groups were calculated using the Kruskal–Wallis’ test, the Pearson’s Chi-square test, or the matched-pair analyses (Wilcoxon signed rank test) and data were presented as median and ranges. Values of *p* ≤ 0.05 were considered significant. No corrections for multiple testing were done.

## Results

Three patients were excluded from the study because routine pathology reports revealed a lack of adenocarcinoma tissue; two patients had an obstruction related to diverticulitis, one had a squamous epithelial cancer. Furthermore, during analysis of blood samples, it was discovered that one patient had received a LV dose that was not according to the protocol and, as a consequence, this patient was excluded from the study. Due to surgical complication with extensive bleeding during the operation, it was not possible to remove the tumour of one patient at the primary operation. This resulted in a time span between the LV injection and tissue collection of almost 30 h. Due to the extended time, the tissue values of folates were not reliable and the patient was excluded from the study. Thus, in total, 75 patients were included. Based on clinical diagnosis, 38 patients had colon cancer and 34 had rectal cancer. Three patients had cancer in both rectum and colon synchronously. The median time (min–max) that passed from LV injection to tissue sampling was 170 min (65–285) for patients receiving 60 mg/m^2^, 165 min (72–457) for patients receiving 200 mg/m^2^, and 163 min (65–555) for patients receiving 500 mg/m^2^. The variation was linked to the operating time, which differed depending on the type of surgical procedure. However, there was no significant difference in the time course from LV injection to tissue sampling between treatment groups (*p* = 0.91). The demographic, clinical and pathological data are shown in Table 1.

**Table 1:** Clinicopathological characteristics of the patients with colorectal cancer sub-grouped by leucovorin dose

Parameter	All patients	Leucovorin dose			
		0 mg/m^2^	60 mg/m^2^	200 mg/m^2^	500 mg/m^2^
Median age in years (range)	72 (37–89)	73 (67–81)	66 (42–87)	70 (37–89)	75 (37–87)
Sex (*n*)					
Men	39	11	12	7	9
Women	36	7	6	12	11
Tumour location (*n*)					
Colon (right/left)	38	10 (7/3)	10 (5/5)	8 (7/2)	10 (7/4/1)
Rectum	34	7	8	10	9
Synchronous	3	1	0	1	1
Tumour stage (*n*)					
I	4	0	0	1	3
II	32	7	7	9	9
III	33	9	9	8	7
IV	6	1	1	2	2
Tumour differentiation (*n*)					
Well	1	1	0	0	0
Moderate	50	14	13	11	12
Poor	17	3	2	6	6
Mucinous	7	0	3	2	2
Pre-operative radio- or chemotherapy (*n*)					
Short-term RT (5 × 5G)	13	0	6	5	2
Long-term RT and CT	3	0	1	1	1

*RT* radiotherapy, *CT* chemotherapy

**Fig. 1:**
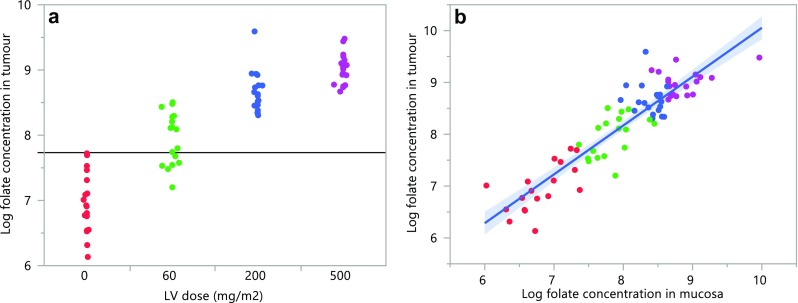
**a** The folate concentration increased with increasing LV dosage in tumour tissue. The horizontal line marks the highest folate concentration found in untreated patients. **b** A significant correlation was seen between folate concentrations in tumour and mucosa of untreated patients (red dots, no LV, *r* = 0.63, *p* = 0.0048) and patients treated with 60 mg/m^2^ LV (green dots, *r* = 0.57, *p* = 0.011), however, the correlation was not significant in patients treated with 200 (blue dots) or 500 (purple dots) mg/m^2^ LV. The line of fit is shown with the confidence interval (blue-shaded area)

The ranges of the tissue folate concentration were very wide, reflecting a huge inter-individual variation (Table 2). The median folate concentration in both tumour and mucosa tissue increased with increasing dosage of LV (Fig. 1a; Table 2). As shown in Fig. 1a, about 50% of patients who received 60 mg/m^2^ did not reach a folate concentration in tumours above the concentration found in untreated patients. There was a strong and significant correlation between the folate concentration in tumour and mucosa tissue of untreated patients, as well as in patients treated with 60 mg/m^2^ LV (Fig. 1b), but no correlation was found in patients treated with 200 or 500 mg/m^2^ LV.

**Table 2:** Comparison of median folate concentration and gene expressions levels in tumour and mucosa tissues of patients with CRC sub-grouped by leucovorin dose

	Leucovorin dose							
	0 mg/m^2^ median (range)	*p* ^a^	60 mg/m^2^ median (range)	*p* ^a^	200 mg/m^2^ median (range)	*p* ^a^	500 mg/m^2^ median (range)	*p* ^a^
Folate concentration^b^								
Tumour	1009 (461–2256)	0.12	3297 (1341–4938)	0.12	5526 (4048–14,641)	0.0079	8248 (5824–13,086)	0.13
Mucosa	847 (413–1591)		2519 (1571–4670)		4576 (2869–5607)		6382 (4487–21,249)	
ABCC3/MRP3								
Tumour	1.7 (0.15–5.1)	0.056	0.90 (0.19–3.3)	0.0002	0.93 (0.12–8.5)	0.0056	1.8 (0.38–9.0)	0.083
Mucosa	2.4 (0.14–43)		2.8 (0.26–15)		3.3 (0.98–7.0)		3.3 (0.80–12)	
FPGS								
Tumour	5.3 (1.1–25)	0.25	5.3 (1.8–30)	0.0021	5.5 (1.0–127)	0.0056	14 (1.2–150)	< 0.0001
Mucosa	3.6 (0.56–318)		2.4 (1.1–11)		3.1 (0.53–29)		3.8 (0.68-48)	
GGH								
Tumour	7.6 (0.35–33)	0.021	4.6 (1.3–22)	0.0021	4.4 (1.5–337)	0.0003	8.4 (0.41–292)	0.0014
Mucosa	1.9 (0.11–49)		2.2 (0.69–7.5)		1.7 (0.37–6.6)		2.4 (0.072–6.9)	
MTHFD1L								
Tumour	1.7 (0.32–9.6)	0.0034	1.5 (0.25–6.2)	0.0008	2.3 (0.33–71)	< 0.0001	2.6 (0.15–19)	< 0.0001
Mucosa	0.44 (0.074–9.2)		0.46 (0.094–7.2)		0.35 (0.047–3.8)		0.41 (0.059–5.6)	
SLC19A1/RFC-1								
Tumour	1.5 (0.33–8.5)	0.47	1.6 (0.17–7.4)	0.011	2.2 (0.42–103)	0.0001	4.4 (0.43–54)	0.0003
Mucosa	0.9 (0.28–11)		0.72 (0.23–4.9)		0.86 (0.17–6.3)		0.96 (0.3–28)	
SLC46A1/PCFT								
Tumour	0.88 (0.02–6.0)	0.13	0.40 (0.062–2.7)	0.034	0.46 (0.08–26)	0.77	1.57 (0.09–40)	0.45
Mucosa	1.1 (0.08–19)		0.65 (0.23–11)		1.1 (0.07–4.1)		1.7 (0.12–8.3)	

^a^ *p* by Wilcoxon signed rank test

^b^pmol/mg

The median concentrations of LV and 5-MTHF in plasma of patients grouped by given LV dose are presented in Table 3. No correlation was found between LV and 5-MTHF in samples obtained from patients who received 60 mg/m^2^ LV (*r* = 0.36, *p* = 0.13). However, there was a strong, positive, correlation between the LV and 5-MTHF concentration in plasma obtained from patients who received 200 or 500 mg/m^2^ LV (*r* = 0.60, *p* = 0.0061 and *r* = 0.76, *p* = 0.0001, respectively).

**Table 3:** Leucovorin and 5-MTHF levels in plasma of patients with CRC sub-grouped by leucovorin dose

	Leucovorin dose			
	0 mg/m^2^ median (range)	60 mg/m^2^ median (range)	200 mg/m^2^ median (range)	500 mg/m^2^ median (range)
Leucovorin^a^				
0 min	0	0	0	0
10 min	0	69.2 (25.8–194)	152.1 (84.3–299)	368 (94.4–918)
30 min	0	45.8 (16.6–114)	111 (72.2–255)	279.1 (61.4–687)
5-MTHF^b^				
0 min	7.32 (0.41–51.0)	6.65 (0.055–91.9)	13.4 (1.23–46.9)	6.07 (0.34–43.8)
10 min	5.98 (0.4–44.3)	240 (81.3–412)	395 (188–1102)	705 (146–1276)
30 min	6.02 (0.45–17.6)	653 (274–1157)	1237 (621–3601)	1909 (538–2914)

^a^nmol/ml

^b^pmol/ml

When the association between 5-MTHF concentration in plasma at baseline and folate concentration in mucosa was evaluated, a positive correlation was found (*r* = 0.5, *p* = 0.035). The maximum plasma concentration achieved for LV was found 10 min. after LV injection whereas the highest 5-MTHF was found after 30 min. Based on these values, the 5-MTHF/LV ratio was calculated for the patients of each group treated with LV (Supplementary Fig. 1). As shown, the ratio between 5-MTHF and LV decreased with higher dose of LV given and a great variation was seen between patients, especially in those who received 60 mg/m^2^. The 5-MTHF/LV ratio correlated negatively with folate concentration in mucosa (*r* = − 0.75, *p* = 0.0009). In patients treated with 200 or 500 mg/m^2^, however, the ratio correlated positively with folate concentration in tumour tissue (*r* = 0.51, *p* = 0.031 and *r* = 0.52, *p* = 0.019, respectively).

Gene expression was analysed in both tumour and mucosa tissues (Table 2). Similar to the folates, the variation in gene expression levels was very high. In untreated patients, the expression of GGH and MTHFD1L was significantly higher in tumour tissue compared to mucosa. After LV injection, a significantly higher gene expression level of GGH, MTHFD1L, SLC19A1/RFC-1, and FPGS was seen in tumour tissue compared to mucosa. However, this was not the case for ABCC3/MRP3 and SLC46A1/PCFTPCFT, which had higher expression levels in mucosa compared to tumour.

**Fig. 2:**
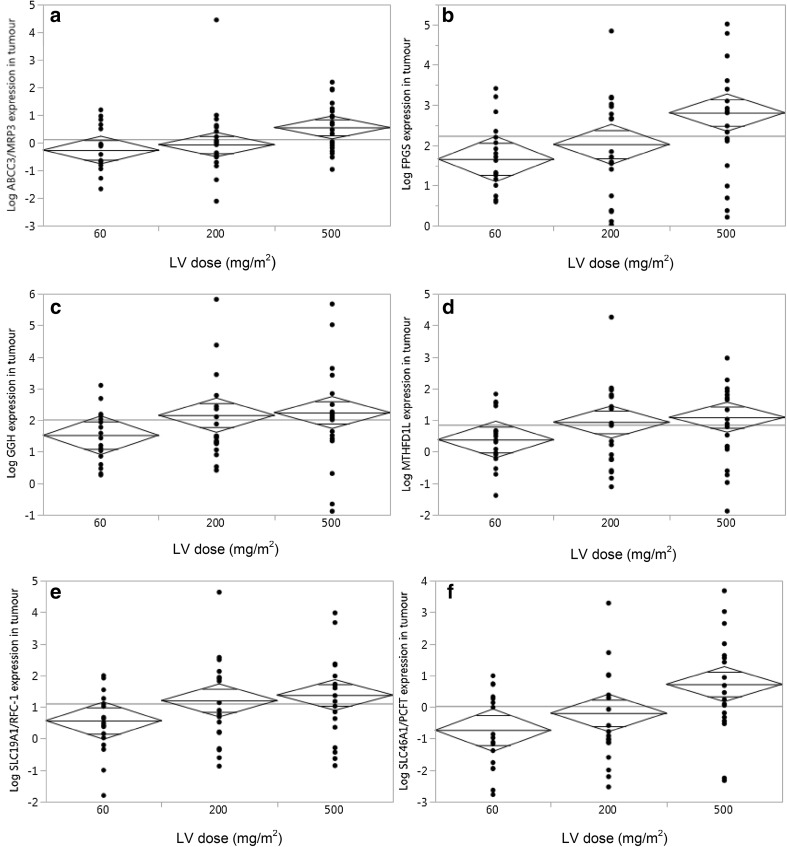
Mean diamonds showing the difference in expression level of **a** ABCC3/MRP3 (*p* = 0.015), **b** FPGS (*p* = 0.044), **c** GGH (*p* = 0.05), **d** MTHFD1L (*p* = 0.18), **e** SLC19A1/RFC-1 (*p* = 0.047), and **f** SLC46A1/PCFT (*p* = 0.011) in tumour tissue of each group. The p values are based on the difference between patient groups treated with the lowest and highest doses of LV, i.e. 60 and 500 mg/m^2^. As shown, the expression increased with increasing dose of LV. The horizontal line in the centre of each diamond shows the mean of each group. The top and bottom points of the diamonds show the upper and lower 95% confidence points

The expression of each gene increased with increasing dose of LV in tumour tissue after adjustment for time passed after LV injection (Fig. 2). However, there was no difference in expression levels according to the LV dose in mucosa (data not shown). The association between folate concentration and expression of each analysed gene in tumour and mucosa was evaluated (Table 4). A significant, negative correlation was seen between folate concentration and GGH and MTHFD1L expression in mucosa of untreated patients, but no correlation was seen in the tumour. In contrast, a significant, positive correlation was found between folate concentration and expression of all genes, except MTHFD1L, in patients who received LV (Table 4). In mucosa, the folate concentration correlated positively with expression of SLC46A1/PCFT.

**Table 4:** Pairwise correlation of gene expression and folate concentration in tissue obtained from untreated patients and patients receiving 60, 200, or 500 mg/m^2^ of LV

Gene	Folate concentration (no LV given)				Folate concentration (60, 200 or 500 mg/m2 LV given)			
	Tumour		Mucosa		Tumour		Mucosa	
	*r* ^a^	*p* ^b^	*r* ^a^	*p* ^b^	*r* ^a^	*p* ^b^	*r* ^a^	*p* ^b^
ABCC3/MRP3	0.13	0.6	− 0.36	0.14	0.32	0.018	0.15	0.28
FPGS	0.32	0.19	− 0.38	0.12	0.31	0.022	0.22	0.11
GGH	0.10	0.69	− 0.52	0.026	0.28	0.041	0.22	0.12
MTHFD1L	0.24	0.35	− 0.50	0.033	0.24	0.078	− 0.027	0.85
SLC19A1/RFC-1	0.32	0.20	0.20	0.43	0.32	0.017	0.21	0.12
SLC46A1/PCFT	0.15	0.56	− 0.44	0.071	0.35	0.0099	0.31	0.024

^a^ *r* = correlation coefficient

^b^ *p* by Pearson

As shown in Table 5, there was a negative correlation between 5-MTHF concentration in plasma obtained from untreated patients and expression of GGH and SLC46A1/PCFT in tumour tissue, but no correlation was seen for any of the other genes. In mucosa of untreated patients, the 5-MTHF concentration tended to correlate negatively with SLC46A1/PCFT.

**Table 5:** Pairwise correlation of gene expression in tissue and 5-MTHF levels in plasma obtained from untreated patients

Gene	5-MTHF in plasma			
	Tumour		Mucosa	
	*r* ^a^	*p* ^b^	*r* ^a^	*p* ^b^
ABCC3/MRP3	− 0.12	0.63	− 0.21	0.39
FPGS	− 0.37	0.13	− 0.36	0.15
GGH	− 0.48	0.045	− 0.23	0.36
MTHFD1L	− 0.30	0.23	− 0.33	0.18
SLC19A1/RFC-1	− 0.44	0.07	− 0.41	0.09
SLC46A1/PCFT	− 0.513	0.03	− 0.45	0.059

^a^ *r* = correlation coefficient

^b^ *p* by Pearson

## Discussion

As previously stated, bolus treatment with 5-FU is given in combination with LV, which significantly enhances the therapeutic effect of 5-FU. FLV-treatment, both in the adjuvant and palliative setting, is usually combined with other chemotherapy drugs such as oxaliplatin or irinotecan, as well as specific antibodies. However, many patients do not respond to the chemotherapy, or will develop drug resistance during treatment. The inter-individual variation in treatment response may relate to differences in drug metabolism [21]. Although 5-FU-based treatment is a cornerstone in almost all CRC regimens, there is no clinically useful biochemical predictive marker that can be used to evaluate treatment effect.

As mentioned previously, LV has to be metabolized intracellularly to the active metabolite 5,10-MeTHF. The optimal intracellular concentration of folates for effective TS inhibition is presently not known but is expected to depend on the individual TS activity as well as the activity of several folate-associated enzymes. In order to get a complete inhibition of the target enzyme TS, 5,10-MeTHF must be in excess. Consequently, a low 5,10-MeTHF concentration in the tumour tissue is associated with clinically impaired 5-FU activity. High doses of LV are needed to achieve a maximum formation of the ternary complex between TS, FdUMP, and 5,10-MeTHF. LV is a racemic mixture of the natural (S) and unnatural (R) diastereoisomers of 5-formyltetrahydrofolate. In contrast to the natural isomer, which disappears rapidly from plasma, the unnatural form has a much longer half-life [12]. High concentration of the unnatural isomer can interfere with the transport and metabolism of the natural isomer. Both of the isomers can interact with TS, and inhibit its activity and thereby also the effectiveness of 5-FU chemotherapy [22].

The results of the present study showed that there was a high inter-individual variation of folates in both tumour and mucosa. This finding is in agreement with previous results published by our group [18] as well as by others [23]. Higher concentrations were found in tumour tissue compared to mucosa in all patient groups. The folate concentration increased with increasing LV dosage in both tumour and mucosa tissue. It is noteworthy that for many patients who received a dose of 60 mg/m^2^ LV, the resulting folate concentration in tumour tissue was as low, or even lower, than the concentration found in untreated patients. This result indicates that some patients may be unable to transport and/or metabolize LV properly. The low tissue folate concentration in some patients may also reflect an initial folate deficiency that requires a higher dosage of LV to achieve satisfying concentrations. Other results of the study points in the same direction; there was a strong and significant correlation between folate concentration in tumour and mucosa obtained from untreated patients as well as in those treated with 60 mg/m^2^ LV, but not between tumour and mucosa of patients treated with 200 or 500 mg/m^2^ LV. The lack of correlation in the latter groups may be due to faster saturation of folates in tumour tissue. Differences between patients treated with 60 versus 200 or 500 mg/m^2^ LV were also reflected in plasma folates and were especially apparent when the 5-MTHF/LV ratio was compared between groups. The 5-MTHF/LV ratio might be useful as a surrogate marker for folate concentration in tumour tissue and, thus, clinically relevant.

In a previous retrospective study, the expression of 22 folate pathway genes having possible impact on the metabolism of LV was analysed in tumours deriving from 193 patients with stage III CRC treated with bolus adjuvant FLV chemotherapy [24]. The analysed genes were involved in folate transport, polyglutamation and metabolism. The result showed that high expression of two genes involved in folate transport, SLC46A1/PCFT and SLC19A1/RFC-1, correlated positively with longer disease-free survival of the patients. It was hypothesized that poor response to FLV therapy in some patients was linked to low expression of these genes. In the present study, the expression of SLC46A1/PCFT and SLC19A1/RFC-1, as well as four other folate pathway genes (ABCC3/MRP3, FPGS, GGH, and MTHFD1L) was determined.

The ABCC3/MRP3 gene encodes a protein that belongs to the superfamily of adenosine triphosphate (ATP)-binding cassette (ABC) transporters. ABCC3/MRP3 carries out an outward transport of a variety of molecules, including monoglutamated forms of reduced folates [25]. The enzyme FPGS converts folate monoglutamates to polyglutamates [26]. Polyglutamate forms of 5,10-MeTHF are better stabilizers of the ternary complex with TS and FdUMP. Studies have shown that LV is ineffective if cells are incapable of metabolizing folate to polyglutamate because the ternary complex will dissociate more readily in the absence of these polyglutamates. The enzyme GGH, on the other hand, cleaves folate polyglutamates to monoglutamates [27]. It has been suggested that GGH regulates the intracellular folate concentration [28]. MTHFD1L is another gene that seems to have a critical role in folate cycle maintenance [29]. The gene encodes a mitochondrial enzyme that catalyses the conversion of 10-formylTHF to THF and formate [30]. Studies have shown that an increased level of MTHFD1L may support colorectal cancer growth [31].

The results of the study showed that tumour tissue of untreated patients had higher expression of FPGS, GGH, MTHFD1L, and SLC19A1/RFC-1 compared with mucosa, whereas expression of ABCC3/MRP3 and SLC46A1/PCFT was higher in mucosa compared with tumour. These results are in agreement with those of our previous study with patients with stage III CRC, where tumour and mucosa tissue were obtained at primary surgery, before onset of adjuvant treatment [24]. Higher expression of ABCC3/MRP3 in mucosa compared with tumour tissue has also been reported by Kobayashi et al. at both the mRNA and protein level [32]. For each of the analysed genes, the expression in tumour increased with increasing dose of LV. Such an increase was not seen in mucosa. After LV injection, the expression of FPGS, GGH, MTHFD1L, and SLC19A1/RFC-1 was significantly higher in tumour tissue compared to the mucosa. However, this was not the case for ABCC3/MRP3 and SLC46A1/PCFT, which consistently had higher expression levels in mucosa, compared to tumour in each of the treatment groups.

Several previous studies have shown that folate deficiency results in a significant upregulation of folate transport genes such as SLC19A1/RFC-1 and SLC46A1/PCFT [33, 34]. Folate deficiency also results in decreased half-lives of these two genes [35]. In the present study, no correlation between gene expression and folate concentration was found in tumour tissue of untreated patients; however, the gene expression levels of GGH and SLC46A1/PCFT in tumour were negatively correlated with 5-MTHF in plasma (i.e. low folates correlated with high expression). Furthermore, high GGH and MTHFD1L expression was associated with low folate concentration in mucosa of untreated patients. This negative correlation may reflect a folate deficiency. After LV treatment, a significant positive correlation was seen between the folate concentration and expression of all genes, except MTHFD1L, in tumour tissue. However, only SLC46A1/PCFT gene expression correlated with folate concentration in mucosa. Studies have shown that the expression level of folate-associated genes in tumour tissue are different compared to mucosa. This disparity may relate to the folate status but also to differences in the regulation of folate metabolism, as has been suggested by Sadahiro et al. [36].

There are limitations of the present study. Firstly, the number of patients included in each group was limited, which may have affected the statistical calculations. However, similar results have been obtained previously in a large and unrelated study, at least at the gene expression level, which strengthens the results. Secondly, tissue was only obtained at one occasion for each of the patients. Thus, no baseline values could be compared with values after LV treatment at the individual level. A larger study is now being planned where samples will be obtained from each patient before and after treatment with LV in combination with 5-FU. Thirdly, the time span for sampling of plasma 5-MTHF was too short to detect the peak level of this metabolite. In the planned study, plasma samples will be obtained during a longer time span in an attempt to detect the peak concentration. Fourthly, the time to tissue sampling after LV injection differed between patients, however, as the time to injection was known, it could be adjusted for in the statistical calculations.

## Conclusions

In summary, there was a high inter-individual variation in tissue and plasma folate concentration in response to administration of LV among patients. Half of the patients who received 60 mg/m^2^ LV did not reach a folate concentration in tumour tissue above the level of untreated patients. Thus, a large part of patients with CRC may benefit from higher LV doses than recommended, or treatment with folate forms with different metabolic profiles. The fact that a strong correlation between mucosa and tumour tissue was found suggests that there is a possibility to use the remnant colorectal mucosa after surgery as a surrogate for tumour tissue during chemotherapy treatment. Prediction of the folate concentration in tissue before treatment with LV would be valuable in order to identify patients who would benefit from higher doses than commonly used. The results further indicate that it might be possible to use the individual response, measured as the ratio between 5-MTHF and LV concentrations in plasma after LV injection, as a surrogate marker for the folate concentration in the target tissue. In the CRC metastatic setting, the possibility to analyse a blood sample would be a useful tool for individualization of folate-based treatment. Analysis of folate-associated gene expression in tissue obtained either from primary tumours, or metastatic lesions in a palliative setting, might become useful when choosing the most optimal LV dose needed to maximize tissue concentration of 5,10-MeTHF. However, the results need to be confirmed in extended studies.

## Electronic supplementary material

Below is the link to the electronic supplementary material.

ESM1 (DOCX 41 kb)
