# Supplementary material for: Relationship between folate concentration and expression of folate-associated genes in tissue and plasma after intraoperative administration of leucovorin in patients with colorectal cancer
Source: Cancer Chemother Pharmacol. 2018 Sep 29;82(6):987–97. doi: 10.1007/s00280-018-3690-9 (PMC6267663; doi:10.1007/s00280-018-3690-9)
Supplement: Supplementary file 1 — ESM1 (DOCX 41 kb) [file 280_2018_3690_MOESM1_ESM.docx]

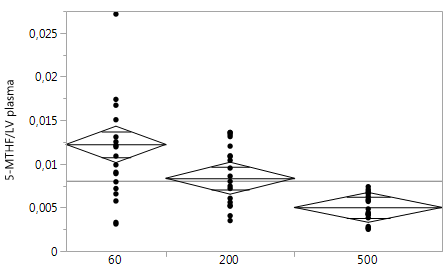


LV dose (mg/m^2^)

**Supplementary Fig. 1** Mean diamonds showing the ratio between 5-MTHF and LV concentrations in plasma measured at 10 and 30 minutes, respectively, in patient groups treated with 60, 200 or 500 mg/m^2^. A significant difference in the mean ratio was seen when the groups were compared (p <0.0001). The horizontal line in the centre of each diamond shows the mean of each group. The top and bottom points of the diamonds show the upper and lower 95% confidence points.
